# Exploring YouTube’s Recommendation System in the Context of COVID-19 Vaccines: Computational and Comparative Analysis of Video Trajectories

**DOI:** 10.2196/49061

**Published:** 2023-09-15

**Authors:** Yee Man Margaret Ng, Katherine Hoffmann Pham, Miguel Luengo-Oroz

**Affiliations:** 1 UN Global Pulse New York, NY United States; 2 Department of Journalism & Institute of Communications Research University of Illinois at Urbana-Champaign Champaign, IL United States; 3 Biomedical Image Technologies, ETSI Telecomunicación, Universidad Politécnica de Madrid & CIBER-BBN, ISCIII Madrid Spain

**Keywords:** algorithmic auditing, antivaccine sentiment, crowdsourcing, recommendation systems, watch history, YouTube

## Abstract

**Background:**

Throughout the COVID-19 pandemic, there has been a concern that social media may contribute to vaccine hesitancy due to the wide availability of antivaccine content on social media platforms. YouTube has stated its commitment to removing content that contains misinformation on vaccination. Nevertheless, such claims are difficult to audit. There is a need for more empirical research to evaluate the actual prevalence of antivaccine sentiment on the internet.

**Objective:**

This study examines recommendations made by YouTube’s algorithms in order to investigate whether the platform may facilitate the spread of antivaccine sentiment on the internet. We assess the prevalence of antivaccine sentiment in recommended videos and evaluate how real-world users’ experiences are different from the personalized recommendations obtained by using synthetic data collection methods, which are often used to study YouTube’s recommendation systems.

**Methods:**

We trace trajectories from a credible seed video posted by the World Health Organization to antivaccine videos, following only video links suggested by YouTube’s recommendation system. First, we gamify the process by asking real-world participants to intentionally find an antivaccine video with as few clicks as possible. Having collected crowdsourced trajectory data from respondents from (1) the World Health Organization and United Nations system (n_WHO/UN_=33) and (2) Amazon Mechanical Turk (n_AMT_=80), we next compare the recommendations seen by these users to recommended videos that are obtained from (3) the YouTube application programming interface’s *RelatedToVideoID* parameter (n_RTV_=40) and (4) from clean browsers without any identifying cookies (n_CB_=40), which serve as reference points. We develop machine learning methods to classify antivaccine content at scale, enabling us to automatically evaluate 27,074 video recommendations made by YouTube.

**Results:**

We found no evidence that YouTube promotes antivaccine content; the average share of antivaccine videos remained well below 6% at all steps in users’ recommendation trajectories. However, the watch histories of users significantly affect video recommendations, suggesting that data from the application programming interface or from a clean browser do not offer an accurate picture of the recommendations that real users are seeing. Real users saw slightly more provaccine content as they advanced through their recommendation trajectories, whereas synthetic users were drawn toward irrelevant recommendations as they advanced. Rather than antivaccine content, videos recommended by YouTube are likely to contain health-related content that is not specifically related to vaccination. These videos are usually longer and contain more popular content.

**Conclusions:**

Our findings suggest that the common perception that YouTube’s recommendation system acts as a “rabbit hole” may be inaccurate and that YouTube may instead be following a “blockbuster” strategy that attempts to engage users by promoting other content that has been reliably successful across the platform.

## Introduction

### Overview

Throughout the pandemic, there has been a concern that the increasing use of social media may contribute to vaccine hesitancy due to the wide availability of antivaccine content on social media [1]. Social media platforms have been blamed for their failure to address the challenge posed by health-related disinformation. The challenge is due partly to the sheer scale of user-generated content and partly to the deployment of recommendation algorithms. These web-based platforms decide what content stays up and what is taken down or blocked, which raises questions such as: How can we balance freedom of expression with the regulation of disinformation? Should we leave the responsibility for regulation and removal of disinformation in the hands of big tech, or would that lead to the privatization of free speech adjudication?

Among social media platforms, YouTube, the most popular video sharing platform, plays a critical role given that users spend around 250 million hours on the platform every day [2]. Past scholarship has noted how influential YouTube can be in shifting individuals’ worldviews over longer periods of time and argued that it may nudge users toward conspiracy theories and antivaccine beliefs [3]. In response, YouTube has stated its commitment to removing “content that falsely alleges that approved vaccines are dangerous and cause chronic health effects, claims that vaccines do not reduce transmission or contraction of the disease, or contains misinformation on the substances contained in vaccines” [4]. Nevertheless, such claims are difficult to audit. One reason is the lack of access to platform data; another reason is the lack of transparency around how its recommendation system operates.

Motivated by the pressing need to mitigate the spread of antivaccine content, we describe a series of approaches to trace a trajectory from a seed World Health Organization (WHO) video to an antivaccine video linked by YouTube’s recommendation system. We set out to answer the following two research questions:

Research question 1: Does YouTube’s recommendation algorithm contribute to spreading antivaccine sentiments on the internet?

Research question 2: What are the proportions of provaccine, antivaccine, neutral, and irrelevant content at each stage of the trajectory through YouTube’s recommender system? How are these proportions affected by the user’s watch history?

Taken together, these questions seek to understand how YouTube’s recommendation system guides users through its content, and to determine the extent to which the platform itself might encourage vaccine hesitancy even among users who are initially seeking legitimate information on vaccines. Such understanding is critical to the public health response to the COVID-19 pandemic, but also carries broader implications for organizations like the WHO, which aim to use YouTube as a tool for disseminating accurate information.

### How YouTube Recommends Videos

To understand whether and how YouTube’s recommender system might encourage vaccine hesitancy, it is first helpful to understand how YouTube recommends videos. The primary purpose of YouTube’s personalized recommendations is to maximize user count, watch time, and retention. The site’s personalized recommendation system is responsible for more than 70% of users’ time spent watching videos on the platform [5]. According to the Pew Research Center [6], over 80% of users watch the videos recommended by YouTube, and YouTube tends to encourage users to watch progressively longer and more popular content.

User behavior, in addition to topically related videos or videos that are often watched together, is a crucial input for YouTube’s personalized recommendations [7]. YouTube’s proprietary recommendation algorithm relies on a deep neural network based on thousands of parameters to suggest videos tailored to individuals [8], including a mix of personal (the users’ demographics, history, and preferences), performance (the video’s engagement and satisfaction), and external (topic interest, market competition, and seasonality) factors; the weighting assigned to each parameter is determined dynamically and varies over time. Although YouTube’s personalized recommendation system has successfully lifted watch time across the site, there have been accusations that such an inscrutable system leads users to misinformation [9] or facilitates extremist content pathways [10,11].

### Down the Rabbit Hole?

The YouTube recommender system aims to direct users to videos that they otherwise might not have selected [12]. However, the role played by the recommendation algorithm in unwittingly promoting misinformation and conspiracy theories is not entirely understood.

On the one hand, some scholars have compared YouTube’s recommendation algorithm to the rabbit hole in “Alice’s Adventures in Wonderland,” a metaphor that refers to its alleged role in steering users to disorienting and disturbing videos [10,13]. For instance, based on users’ comments across communities and across time, Ribeiro et al [11] documented a sizable migration from right-wing contrarian channels to fringe far-right channels, illustrating the existence of radicalization pathways on YouTube. On the other hand, some scholars have warned against overestimating the impact of YouTube’s recommendations. Munger and Phillips [14] argued that most scholarship adopted a “Zombie Bite model of YouTube radicalization,” which downplayed users’ agency and other platforms’ affordances. They argued that YouTube was not radicalizing an otherwise moderate audience, but rather acting as a content supplier to create radical alternative political canons and interpretive communities to match an ideology that already exists. Ledwich and Zaitsev [15] even argued that, if anything, YouTube’s recommendation engine had a deradicalizing influence by favoring mainstream media over independent channels. Ribeiro et al [11] highlighted that it is challenging to make conclusions on YouTube’s role in radicalization using data from anonymous recommendations since the radicalization hypothesis concerns personalized recommendations.

### Related Studies on Antivaccine Content

Even before COVID-19, vaccine misinformation on social media led to significant drawbacks in efforts to increase vaccine coverage rates. Past scholarship has studied how prevalent antivaccine content is on social media. On YouTube, early research showed that negative sentiment toward vaccines had been steadily increasing from 32% in 2007 [16] to 51.7% in 2012 [17], and 65.5% in 2017 [18] among videos related to the topic of vaccination. Song and Gruzd [19] studied the importance of antivaccine content in a network of vaccine-related videos and suggested that watching antivaccine content facilitates pathways through the recommender system to more antivaccine content. However, a more recent study by Abul-Fottouha et al [20] found that provaccine videos (64.75%) are more prevalent than antivaccine (19.98%) videos on YouTube. Papadamou et al [21] stated that the recommender system was unlikely to suggest COVID-19 pseudoscientific content in comparison to other types of pseudoscientific content (eg, flat-earth content). Tang et al [22] found that when users searched on YouTube using keywords, regardless of whether they used provaccine or antivaccine keywords, they were likely to reach provaccine videos posted by credible sources such as government agencies and hospitals. These differing results could reflect YouTube’s recent efforts to demonetize harmful content and reduce misinformation, as well as changes to the recommendation algorithm over time.

YouTube itself acknowledges the potential adverse impacts of social media. The platform has updated its policies to reduce harmful antivaccine content that spreads medical misinformation about currently administered, approved vaccines [23]. For example, the site does not allow videos making false claims that vaccines cause chronic side effects, such as cancer or diabetes, or that vaccines do not reduce the risk of contracting an illness. These measures indicate that YouTube is aware of the availability of problematic antivaccine content on its platform.

### Lack of User Activity Data

Auditing YouTube’s personalized recommendation system is challenging. One reason for this is the algorithm’s complex and opaque nature [24], and another is that user activity data are only available to social scientists who work for YouTube. Due to limited access to (and an incomplete understanding of) the YouTube recommender system, the models built by researchers might not reflect the actual mechanisms underlying the YouTube recommender system and may overlook or underestimate pathways to problematic content [11,25].

Earlier research has relied on anonymous, impersonal accounts without any watch history to audit YouTube’s recommendation system. However, to address the lack of user activity data, some researchers have intentionally curated user profiles to simulate personas—distinct demographics, content preferences, and watch histories—in order to generate personalized recommendations [26]. For example, to investigate the effects of personalization on the amount of misinformation in YouTube search results, Hussein et al [27] built a crawler that watches a curated subset of the videos returned by search queries in order to build the watch history of their user profiles. Then, they collected the “Up-Next” and “Top-5” video recommendations to assess how the user profiles affected the videos listed in the recommendations section of the platform. Similarly, Papadamou et al [21] carefully crafted 3 user profiles, each with a different watch history, to emulate logged-in users. They found that the minimum number of videos users need to watch before YouTube learns one’s interests and generates personalized recommendations is 22. Furthermore, they found that YouTube Data application programming interface (API) results were similar to those of the non–logged-in profile with no watch history (using a browser); this indicates that recommendations returned using the API are not subject to personalization.

However, few studies have collected a sizable amount of data for analysis while being signed into YouTube [11,15]. Such a strategy would demand the creation and maintenance of dozens of YouTube profiles—a complex and time-consuming task that researchers have understandably avoided. While Chen et al [28] used a plug-in browser extension as a strategy for recording users’ web history and YouTube viewing choices (in the format of URLs) in a scalable way (859 participants and activity data averaged 64 days), their method raised concerns about collecting too much personal data, and the hyperlink recorded by the extension only revealed which videos users chose to watch, rather than broadly capturing which videos YouTube recommended on the site. To address these shortcomings, this study gathered data on personalized YouTube video recommendations from actual users by collecting HTML files from the YouTube pages they visited. We then compared their recommended trajectories to recommended videos that were obtained from the YouTube API’s *RelatedToVideoId* field and from browsers without any identifying cookies.

## Methods

### Overview

**Figure 1:**
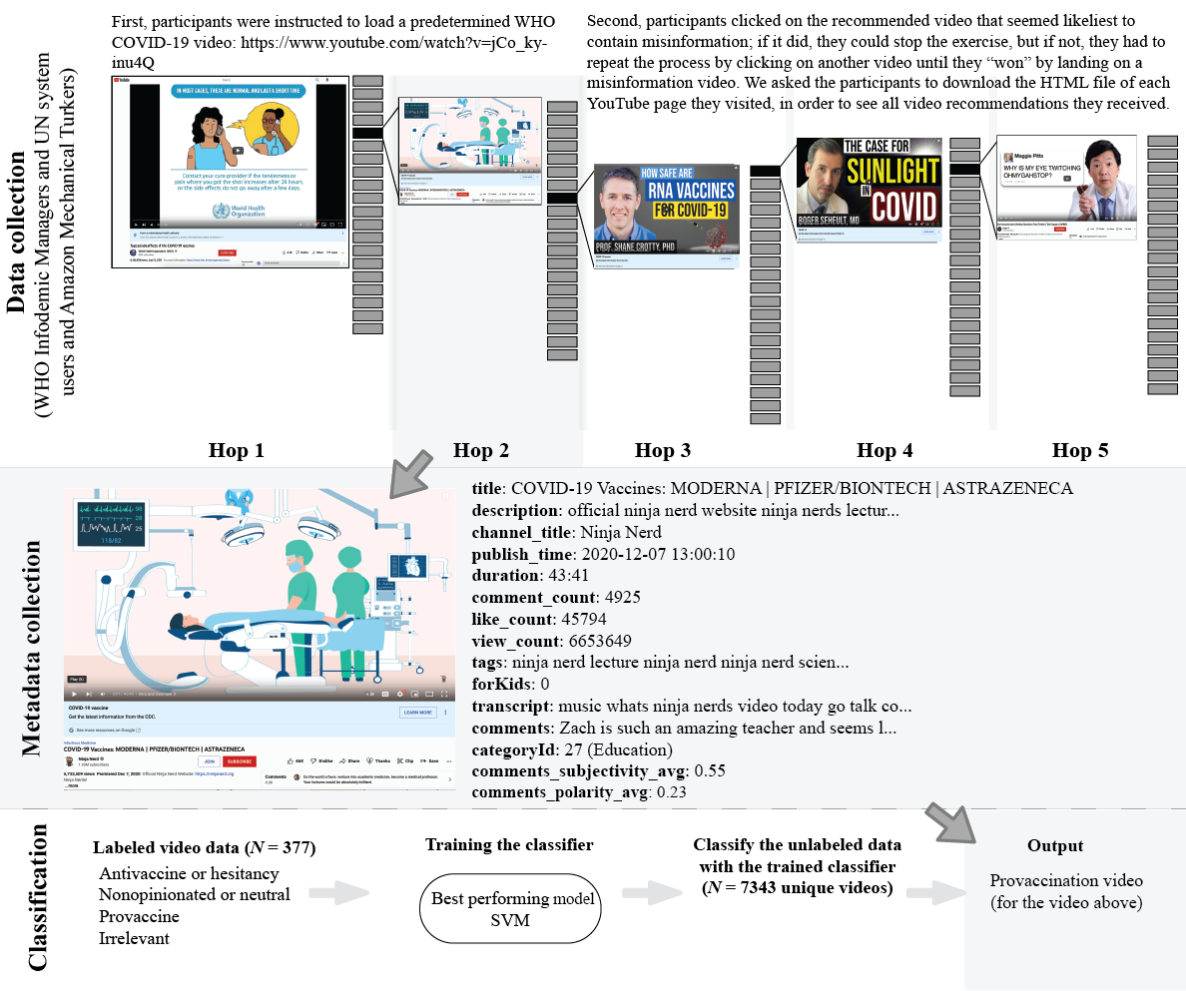
The pipeline for constructing the data set used in analysis, including the data collection process for YouTube data with user watch history, the features collected using the YouTube application programming interface, and the classification process. SVM: support vector machines; UN: United Nations; WHO: World Health Organization.

We collected recommendation trajectories using the following four different approaches: (1) recruiting volunteer participants who enrolled in a training for infodemic managers organized by the WHO; (2) hiring workers from Amazon Mechanical Turk (Mturk); (3) using the YouTube API’s *RelatedToVideoId*; and (4) collecting YouTube’s Up-Next recommended videos using simulated browsers without any identifying cookies. The former 2 approaches allowed us to assess how likely it was for users with distinct watch histories to come across disinformation content on YouTube, while the latter 2—presumably reflecting YouTube’s recommendation mechanism without any personalization—served as reference points. Figure 1 illustrates the data collection process for YouTube data with user watch history, the features collected using the YouTube API, and an example of video classification.

### Recruitment

#### Approach 1 and 2: Data With User Watch History

We recruited members from an infodemic manager training led by the WHO, as well as a convenience sample of additional volunteers from within the United Nations (UN) system assembled through internal social networks, between December 2021 and February 2022. The infodemic manager training participants were typically mid- and senior-level public health professionals who worked within or with governments and were interested in advancing their knowledge of strategies for infodemic management. We also recruited participants from Mturk in February and August 2022. Both groups of participants were instructed to load a predetermined WHO COVID-19 video (see Textbox 1 for details). Then, they were asked to click on the recommended video that seemed likeliest to contain antivaccine or hesitancy content; if it did, they could stop the exercise, but if not, they had to repeat the process by clicking on another video until they “won” by landing on an antivaccine or hesitancy video. We asked the participants to download the HTML file of each YouTube page they visited during this process. We chose to collect HTML files because they preserved the recommendation information to its full extent (at least 20 recommended videos without scrolling down) and were less prone to human input errors. The manual download process could be repeated quickly whenever participants clicked on the next video and loaded a new web page. To minimize data collection effort, we only asked the participants to do this for the first 5 pages they visited; after these first 5 “hops,” we just collected hyperlinks to any subsequent YouTube videos visited until the participants reached an antivaccine video. Finally, we asked participants to explain why they thought the final video contained antivaccine sentiment and to answer basic demographic questions about their age, gender, country, and the primary language they use on the web.

**Information about the seed video.**

Title: “Typical side effects of the COVID-19 Vaccines”

Channel: World Health Organization (WHO)

Number of views: 4,187,178 views (as of October 13, 2022)

Published Date: June 15, 2021

Length: 38 seconds

URL: https://www.youtube.com/watch?v=jCo_kyinu4Q [29]

#### Approach 3: The YouTube API’s RelatedToVideoId

We compared these recommended videos based on user behavior to videos obtained from the YouTube API, which is designed to help programmers search for content on the platform. We collected videos using the *RelatedToVideoId* parameter, which “retrieves a list of videos that are related to the video that the parameter value identifies” [30]. There have been discussions [4] arguing that videos returned from the *RelatedToVideoId* parameter may not always reflect the actual recommendations a user sees. In other words, there are differences, conceptually and algorithmically, between the API’s “related” videos and its “recommended” videos. On YouTube, videos are regarded as “related” based on the interconnectedness of video producers, channels, and videos; their use of similar catchphrases; the user’s own activity data and the activity data of similar users; and covisitation counts [31]. We followed these related-video recommendations to a depth of 10 hops, retrieving at least 20 related videos at each step. We used the first related video returned to progress forward at each step. We repeated this process 40 times to (n_RTV_=40) generate different potential trajectories based on the randomness in what the API returns.

#### Approach 4: YouTube’s Up-Next Recommended Videos

As an alternative to the YouTube API, recent studies have gathered recommended videos using clean browsers because this approach arguably bears a closer resemblance to the user’s experience [11,15]. We took this approach to obtain recommended videos based on the first watch-next recommendations. Using the Python packages Selenium and Beautiful Soup, we activated incognito browsers to replicate the experience of a new user visiting YouTube with no search or view history. The crawling process was automated for 10 hops, collecting 20 recommended videos and following the top recommended video at each step, and then repeated 40 times (n_CB_=40) to generate a set of sample trajectories based on the randomness in what the web page returns.

We collected a total of 27,074 videos recommended in trajectories starting from the seed video using these 4 approaches. Table 1 summarizes our data set. After collecting the data, we parsed each trajectory and collected the metadata of each video using the YouTube Data API.

**Table 1:** Data collection.

Approaches			Locations		Responses, n		Hops, n		Videos, n (% unique)	
**Real-world data with user watch history**										
	WHO^a^ infodemic managers and UN^b^ system	Global		33		5		3652 (46)		
	Amazon Mechanical Turk	United States		80		5		7422 (41)		
**Recommended videos without user watch history and any identifying cookies**										
	YouTube API^c^ (*RelatedToVideoId*)	United States		40		10				8000 (10)
	Simulated browser and Up-Next recommended videos	United States		40		10		8000 (23)		

^a^WHO: World Health Organization.

^b^UN: United Nations.

^c^API: application programming interface.

### Video Classification

#### Overview

After successfully gathering a large volume of YouTube recommendations, we faced another challenge: it is far from trivial to categorize a large number of videos according to their ideological affinity (in our case, their pro- or antivaccine or hesitancy stance). However, there have been promising attempts to use natural language processing to scale up video classification tasks. Therefore, we attempted to address the coding problem by collecting and manually annotating a training set, which we then used to build a classifier that relied on diverse text sources (the video transcript, title, description, tags, comments, etc) and other aspects of the video metadata (such as likes and views) to predict a video’s stance on vaccination.

#### Annotating the Training Data

To create a training data set, we collected 500 videos using targeted keywords (eg, “anti-vax” and “vaccine choice”; see Textbox 2) through the YouTube API. To capture diverse types of videos, we collected the top 125 videos ranked according to each of the following criteria: (1) relevance, (2) creation date (newest to oldest), (3) rating (highest to lowest), and (4) view count (highest to lowest). In case of overlap, we replaced the videos with the next-ranked videos for each criterion.

**Keywords to collect YouTube videos for classification training data.**

**To identify vaccine-related content, we used the following query:**

- vaccine OR vaccines OR vaccina* OR jabs OR “jab” OR antivax OR antivax* OR “no-vax” OR “no-vax*” OR immunisation OR “vaccination” OR vaccine choice) AND (coronavirus OR covid OR “sarscov2” OR “coronavirus” OR “covid19” OR “cov19” OR 19 OR nineteen OR “covid nineteen”

**Antivaccine content could be harder to find. Therefore, we intentionally also added the following additional keywords that leaned toward antivaccine sentiment to expand our search:**

- “my body, my choice,” “pro-choice,” “vaccine causes autism,” “vaccine kills,” “vaccine takes life,” “vaccine harm,” “vaccine choice,” “severe illness after vaccination”

We presented each video to 4 annotators recruited from MTurk. Previous scholarship has indicated that MTurk workers complete categorization tasks and crowdsourced content analysis with reliability and accuracy that are comparable to both students and expert raters [32]. We asked MTurk workers to inspect the video content and apply one of the following four labels to each video:

1. Antivaccine or hesitancy (–1): The primary intention of the video is to explain reasons not to get the COVID-19 vaccine, to delay acceptance, to encourage people to refuse vaccination despite availability, to oppose vaccination mandates, to support vaccine choice, or to inspire skepticism about the safety of vaccination (eg, to suggest that the vaccine may contain heavy metals or could result in long-term side effects such as autism or infertility).
2. Nonopinionated or neutral (0): The video contains only factual information (eg, the COVID-19 vaccine is authorized for children aged 5-11 years) and news (not commentary) that objectively discusses vaccination rates, protests, lawsuits against vaccine mandates, or other aspects of policy implementation. Pro- and antivaccine positions are represented equally.
3. Provaccine (+1): The primary intention of the video is to promote vaccination as the primary way to put the pandemic behind us, to debunk antivaccine myths, to explain vaccine ingredients and the mechanisms by which vaccines build immunity, to feature vaccine advocates and vaccine providers, to share (nonnegative) personal vaccination experiences, to outline the vaccination process, or to explain the vaccine’s side effects in an educational manner.
4. Irrelevant (999): The video is not related to vaccination. For example, lo-fi music and video game videos are considered irrelevant.

Table 2 presents the definitions and example videos for each of these different categories. Only videos for which at least three of four annotators agreed on the label were included in the data set (377/500, 75%). Those videos were then split into a training set (340/377, 90%) and a test set (37/377, 10%). The training set was used to build the classification model, and the performance was evaluated on the test set.

**Table 2:** Video classification examples.

Categories and definition			Examples
**Antivaccine or hesitancy**			
	Explain reasons for not getting COVID-19 vaccine; encourage delay in acceptance or refusal of vaccines; etc	COVID-19: GP explains why she won’t have vaccine but says she’s not anti-vax (Sky News) COVID-19 Acute Necrotizing Encephalopathy (NeuroscIQ)	
**Nonopinionated or neutral**			
	Factual information; news, not commentary; both pro- and antivaccine stands are equally presented; etc	How coronavirus is changing the world (DW Documentary) How Moderna And Pfizer-BioNTech Developed Vaccines In Record Time (CNBC)	
**Provaccine**			
	Promote vaccination as the primary way to put the pandemic behind us; debunk antivaccine myths; etc	How Do mRNA Vaccines Work? (SciShow) Debunking Anti-Vaxxers (AsapSCIENCE)	
**Irrelevant**			
	Not related to the topic of vaccination	The Truth About 5 Health Food Trends | Compilation (SciShow) Possible new antivirals against COVID-19, herpes (American Chemical Society)	

#### Features

We collected the following data for each of the videos using the YouTube Data API:

1. Numeric features, including video duration, comment or reply count, like count, and view count.
2. Categorical features, including genres and whether the video is for kids.
3. Textual features, including (1) the video snippet: the concatenation of the title, the description, and the tags of the video. The snippet contains the language used by the content creator to describe their video. (2) The transcript of the video: subtitles, which can be uploaded by the creator or autogenerated by YouTube and which capture the audio content of the video. The transcript allows us to also consider the main themes discussed in the actual video, rather than just the language used to describe it on the platform. And (3) the comments on the video: the top 10 comments on the video, ranked by YouTube’s relevance metric. For each video, we combined the 3 textual features described above into one single long line of text and applied natural language processing to clean the data (ie, removal of stop words, tokenization, lemmatization, and removal of special symbols). After these cleaning steps, we converted the text-based features into embeddings. We used term frequency-inverse document frequency (TF-IDF) and pretrained word embeddings created with the global vectors for word representation (GloVe) algorithm [33] to transform the features into vectors representing the data. While TF-IDF relies on a sparse vector representation, GloVe creates dense vector representations. TF-IDF is a measure of word importance that weighs the relative frequency with which a word is used in a given document (TF) against how often the word appears in documents across the entire corpus (IDF). Effectively, it seeks to identify words that set individual documents apart from the general corpus. Table 3 presents the most discriminating words in the training set for video classification, ranked by TF-IDF. On the other hand, GloVe is an unsupervised learning algorithm that puts emphasis on the importance of word-word co-occurrences to extract meaning, as opposed to other techniques such as skip-grams or bag-of-words models. We used pretrained embeddings generated from the Wikipedia corpus, which we adopted to create features for our classification data set.
4. Polarity and subjectivity: in addition to processing the text features, we calculated the sentiment scores of the top 10 comments of each video. Sentiment analysis can help us decipher the mood and emotions of the general public and gather insightful information regarding the context. We used TextBlob [34], an API for automatically processing text data that includes sentiment analysis functionality, to measure polarity and subjectivity. Evaluations in previous literature [35] show its competitive performance on conventional text data sets. The polarity score, with a range from –1 (negative sentiment) to +1 (positive sentiment), captures sentiment intensity based on grammatical and syntactical conventions. Subjectivity ranges from 0 (objective) to 1 (subjective). Objective statements present substantiated factual information, whereas subjective sentences generally refer to opinion, emotion, or judgment.

**Table 3:** Selected top correlated unigrams and bigrams (by term frequency-inverse document frequency) in the training set.

			Antivaccine or hesitancy		Nonopinionated or neutral		Provaccine		Irrelevant
**Video description**									
	Unigrams	passports protest law truckers djokovic		descend pass brandon demonstrators senate		virus leaders medical doctor encourage		teach play life enjoy game	
	Bigrams	television news brand key human right across country novak djokovic		two years booster shots mask mandate also tighten thousands demonstrators		mandatory vaccinations convoy protest omicron variant booster shots wear mask		news world like facebook social media facebook page thank watch	
**Video transcript**									
	Unigrams	socialist legislature gender freedom discriminate		economic surge bustle bridge blockade		risk power dose virus protesters		machine pop ball power game	
	Bigrams	antivaxx rally work around kid face know science time thank		mandate public supply chain police protesters national mall control control		love ones second dose clinical trials protect family health care		phone number joe biden guy guy come come please share	

#### Model Training

To train the model, we tested a mix of traditional machine learning and deep learning models to explore different modeling paradigms and compare their performance, including support vector machines (SVM) [36], Random Forests [37], XGBoost [38], AdaBoost [39], Long Short-Term Memory (LSTM [40], a type of recurrent neural network), and region-based convolutional neural network [41], a type of convolutional neural network (CNN). SVM is a powerful and interpretable classifier, while ensemble methods like Random Forests, XGBoost, and AdaBoost offer robust performance and the ability to capture complex relationships. LSTM and region-based convolutional neural networks are state-of-the-art deep learning models. Recurrent neural networks, including LSTM, are particularly effective in handling sequential data, where the order of words or phrases is crucial for understanding the context. While CNNs are primarily designed for image data, they can also be applied to text data by treating it as a 1D sequence. In this context, CNNs can focus on local patterns within the transcript, such as detecting specific phrases for sentiment analysis or named entity recognition. To get a more robust result, we applied 10-fold cross-validation on the training set.

Our class distribution was highly imbalanced (175/377, 46.4% provaccine; 48/377, 12.7% neutral; 76/377, 20.1% antivaccine; and 78/377, 20.6% irrelevant), even though we intentionally searched for antivaccine content (see Textbox 2 for details). Therefore, we applied the synthetic minority oversampling technique (SMOTE [42]) to the training data. This is a common and proven technique in machine learning to balance the distribution of the categories in a classification problem. It uses oversampling to prevent the classification model from favoring the more frequent category in the case of unbalanced class distribution [43].

### Ethical Considerations

We obtained an institutional review board’s exemption for human participant use (Protocol: 22813, University of Illinois). Participants were informed about the study and provided their consent through a web-based form, which emphasized the voluntary nature of their participation and their right to withdraw at any time. The data sets in this paper include user video browsing information and therefore might have privacy implications. Therefore, all data used in this study were collected following established ethical procedures for social data. We also ensured the data were anonymized and securely stored in a protected environment. Mturk participants were compensated with US $5 for their time.

## Results

### Participants

All of the participants from the infodemic manager training led by the WHO and the UN (n_WHO/UN_=33; aged 18-60 years) spoke English as a first or second language; 23 (69%) of them were female, 9 (28%) were male, and 1 (3%) preferred not to specify their sex. All participants from MTurk (n_AMT_=80; aged >18 years) were English speakers from the United States; 30 (37%) were female and 50 (63%) were male.

Among 33 WHO and UN participants, 29 (88%) did not find an antivaccine video in the first 5 hops, while 3 (12%) reasoned that the videos they last encountered delivered antivaccine or hesitancy messages. Among 80 MTurk workers, 64 (80%) did not find an antivaccine video in the first 5 hops, while 16 (20%) mentioned they landed on a video focused on antivaccine or hesitancy messages. However, many participants were uncertain whether the videos they watched contained antivaccine or hesitancy content or not.

### Model Results and Evaluation

The best classification result was achieved by SVM, which had the best overall accuracy (0.72), recall (0.72), and *F*_1_-score (0.72; *F*_1pro_=0*.*78, *F*_1neutral_=0*.*67, *F*_1anti_=0*.*70; *Recall*_pro_=0*.*70, *Recall*_neutral_=0*.*64, *Recall*_anti_=0*.*79). A high recall is particularly important in this case, as we want to minimize the chance of missing antivaccine or hesitancy videos (predicting false negatives). A high *F*_1_-score is also important for cases where the data set is imbalanced, as it requires both precision and recall to have a reasonable value. Tables 4 and 5 show the performance metrics for classification.

**Table 4:** Performance metrics for classification (with term frequency-inverse document frequency and global vectors for word representation).

Models	Accuracy	Precision	Recall	*F*_1_-score
SVM^a^	0.72	0.74	0.72	0.72
Random Forest	0.66	0.75	0.66	0.66
XGBoost	0.64	0.63	0.64	0.63
AdaBoost	0.38	0.44	0.44	0.40
CNN^b^ (RCNN^c^)	0.52	0.50	0.52	0.51
RNN^d^ (LSTM^e^)	0.58	0.61	0.58	0.54

^a^SVM: support vector machines.

^b^CNN: convolutional neural network.

^c^RCNN: region-based convolutional neural network.

^d^RNN: recurrent neural network.

^e^LSTM: Long Short-Term Memory.

**Table 5:** Overview of the support vector machines classification models.

Textual features	Accuracy	Precision	Recall	*F*_1_-score
TF^a^-IDF^b^	0.68	0.56	0.60	0.61
GloVe^c^	0.60	0.53	0.56	0.59
TF-IDF and GloVe	0.72	0.74	0.72	0.72

^a^TF: term frequency.

^b^IDF: inverse document frequency.

^c^GloVe: global vectors for word representation.

**Figure 2:**
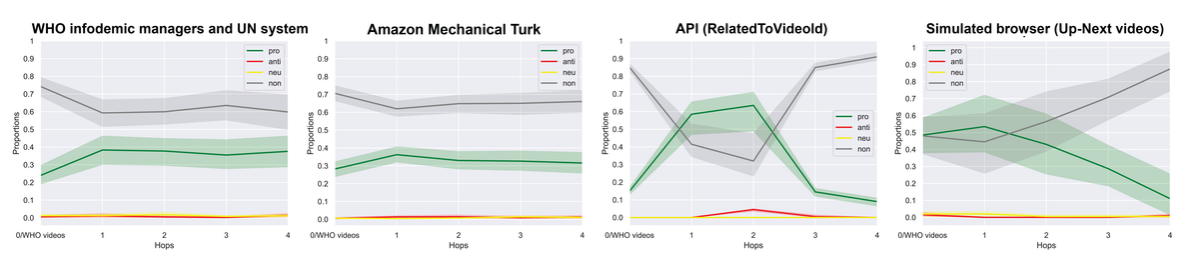
Estimated central tendency and confidence intervals for the first 5 hops. API: application programming interface; UN: United Nations; WHO: World Health Organization.

After determining that our SVM classifier had achieved satisfactory performance, we next used the classifier to label all of the videos in our trajectory data sets. This allows us to compare how the stance of the videos (provaccine, neutral, antivaccine, and irrelevant) varies over the course of individual trajectories and across our 4 different trajectory data sets. Figure 2 plots the proportion of videos for the first 5 hops with each approach.

### How Videos Vary Over the Course of Recommendation Trajectories

When looking at samples of real users (the left 2 panels of Figure 2), we found that the percentage of vaccination-related videos varies depending on what the participants click. Among WHO Infodemic Managers and UN system users, the percentage of provaccine videos recommended alongside the seed video was around 22.5% (149/660 videos at the first hop) and converged at approximately 40% (264/660) as the participants clicked on more recommendations. Participants from MTurk showed a very similar pattern, although they saw a slightly lower share of provaccine videos on average, and a slightly higher share of irrelevant videos. This pattern is contrary to our expectation that users would see a higher share of antivaccine videos as their search for antivaccine content advanced. There could be two possible explanations for this pattern: (1) users are not effective at identifying antivaccine content and are simply finding more vaccine-related content (including positive content) as they search; or (2) YouTube is funneling users toward provaccine content as they search. Regardless, these findings do not support a “rabbit hole” narrative in which users are getting pushed toward more scientifically questionable, radical, and “fringe” content.

**Figure 3:**
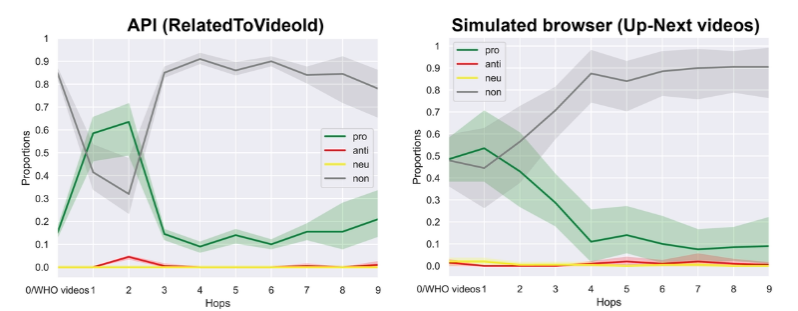
Estimated central tendency and confidence intervals for the first 10 hops. API: application programming interface; UN: United Nations; WHO: World Health Organization.

**Figure 4:**
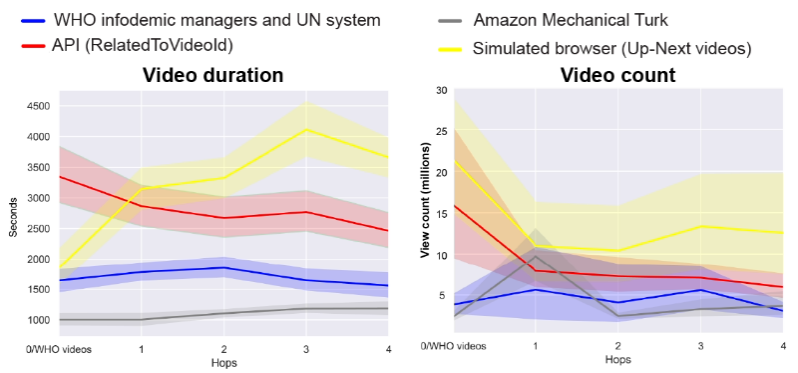
Comparison of video duration and view count for the first 5 hops. API: application programming interface; UN: United Nations; WHO: World Health Organization.

However, the trends look very different when considering data from the YouTube API and simulated browsers (the right 2 panels of Figure 2). These trajectories show large initial fluctuations in the share of content by category, followed by a strong convergence on recommendations of irrelevant content. Looking at the data from full trajectories of 10 hops (Figure 3), the share of irrelevant videos levels off at approximately 90% (720/800 videos of each hop), while the share of provaccine videos drops sharply to approximately 10% (80/800) to 20% (160/800). Simulated browsers show a more steady increase in the share of irrelevant videos to 90% (720/800 videos of each hop) and a steady decrease in the share of provaccine videos to less than 10% (80/800). These results also fail to indicate the presence of a “rabbit hole,” although they do suggest that in the absence of real user watch histories, YouTube is progressively pushing vaccine-interested users toward more and more unrelated content, which is usually longer and more popular content that has already accumulated many views (see Figure 4).

### How Videos Vary by Data Collection Approach

We validated the statistical significance of the differences in the proportions of provaccine videos and irrelevant content suggested to the profiles with watch history as compared to YouTube’s API and simulated browsers through Fisher Exact tests (*P*<.05). This suggests that the data collected through the API and simulated browsers are not an adequate substitute for the recommendation trajectories of real users.

### Analysis of Videos That Are Recommended by YouTube

**Figure 5:**
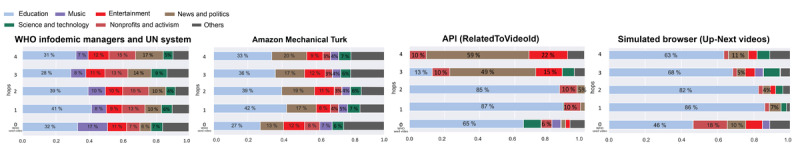
Distribution of YouTube video content by category for the first 5 hops. API: application programming interface; WHO: World Health Organization.

To understand whether the types of recommendations changed over different approaches, we also studied the genre composition change over the first 5 hops (Figure 5), where “genre” is taken from YouTube’s official video categories [44]. Results showed that for real users, the composition of the genres was relatively consistent across hops. In both the WHO, UN, and MTurk samples, education videos were most common, followed by news, politics, and entertainment videos. Among users who were from the WHO and UN samples, nonprofits, activism, and music were also common categories. It is not surprising that the former category is popular among WHO and UN audiences, as the WHO official channel is categorized as nonprofits and activism.

**Figure 6:**
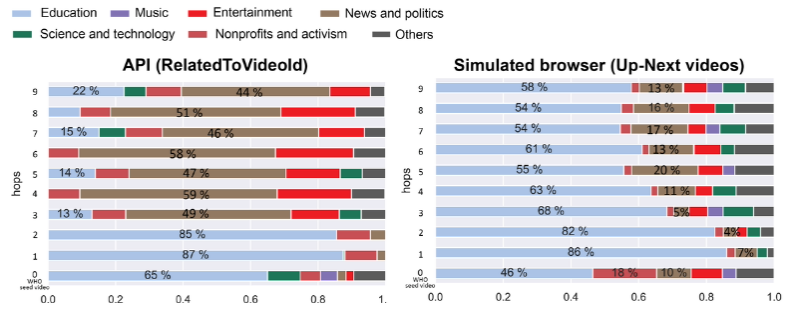
Distribution of YouTube video content by category for the first 10 hops. API: application programming interface; WHO: World Health Organization.

Interestingly, when using the API or a simulated browser, we do see changes in the types of recommendations made over time. In trajectories generated through the API, we observe that YouTube starts with a high share of education videos but gradually pushes more content on news and politics, as well as entertainment. On the other hand, data collection with a simulated browser showed a disproportionately high portion of education content throughout all 10 hops (see Figure 6), although the share of education content did fall gradually over time in favor of other types of content.

Given the large proportion of unrelated content suggested to both real and synthetic users, we next investigated the type of content that appears in this category. Among nonrelated videos, we found that YouTube frequently recommended popular (high view count and from popular channels) medically related videos, addressing topics such as attention-deficit hyperactivity disorder, heart disease, vitamin D deficiency, seizures, or depression (Textbox 3).

**Top 3 frequent irrelevant videos recommended by YouTube.**

**World Health Organization infodemic managers and United Nations system:**

- ADD/ADHD | What Is Attention Deficit Hyperactivity Disorder? (28:14, 8,063,485 views) by Understood, 128K subscribers
- How to recover from depression (1:02:35, 4,350,702 views) by Psychlopaedia.org, 53.3K subscribers
- Sugar: The Bitter Truth (1:29:36, 22,385,869 views) by University of California Television, 1.18M subscribers

**Amazon Mechanical Turk:**

- Living into your 90s - Factors that can lead to a longer, healthier life (25:48, 6,130,751 views) by 60 Minutes, 1.71M subscribers
- Excess deaths, the data - Athletes who die suddenly of heart disease (15:29, 1,224,492 views) by Dr. John Campbell, 2.46M subscribers
- Sugar: The Bitter Truth (1:29:36, 22,385,869 views) by University of California Television, 1.18 M subscribers

**Application programming interface (*RelatedToVideoId*):**

- Humans finally figured out how to make it rain (9:45, 987,076 views) by Vox, 10.9M subscribers
- How “dementia villages” work (7:09, 1,688,901 views) by Vox, 10.9M subscribers
- Vitamins D and K2 (40:21, 2,125,031 views) by Dr John Campbell,
- 2.46M subscribers

**Simulated browser:**

- Seizures | Etiology, Pathophysiology, Clinical Features, Treatment, Complications/Status Epilepticus (1:40:48, 492,127 views) by Ninja Nerd, 1.94M subscribers
- How to Speak - Improve your speaking ability in critical situations (1:03:42, 13,342,436 views) by MIT OpenCourseWare, 4.16M subscribers
- MASTER ECG/EKG INTERPRETATION: A Systematic Approach for 12 Lead ECG/EKGs (59:13, 873,344 views) by Ninja Nerd, 1.94M subscribers

## Discussion

### Overview

This study investigates how YouTube recommends videos in order to better understand how the platform’s artificial intelligence–enabled recommendation system shapes the information users have access to and how it may influence users’ vulnerability to antivaccine or hesitancy information. We leverage the potential of machine learning techniques to classify videos at scale and uncover video recommendation patterns on YouTube over almost 200 recommendation trajectories, which together include over 27,000 suggested videos.

We developed a complete and reusable framework that allows us to assess the prevalence of antivaccine or hesitancy content on YouTube using 4 different approaches to data collection. This allows us to account for the effect of a user’s watch history over different categories of users (a specific class of users recruited from the WHO and UN system, and a more general class of users recruited through MTurk), and to compare the experience of these real users against the recommendations that are more easily retrieved from the YouTube API and using a clean, simulated browser.

### Principal Results

Our findings are as follows. First, this exercise does not support the conclusion that YouTube acts as a “rabbit hole” for radicalization or misinformation, at least in the context of antivaccine sentiment during the COVID-19 pandemic. In trajectories originating from a WHO-posted vaccine video, we find very few recommendations of antivaccine videos and a higher likelihood of converging on provaccine or irrelevant content. If anything, YouTube appears to push users to vaccine-unrelated content on other health topics, similarly to the findings of Tang et al [22], suggesting that it may be driving users into the mainstream (by pushing content that is popular elsewhere on the platform) rather than toward the fringe. This may be a function of YouTube’s commitment to removing content that falsely alleges that approved vaccines are dangerous and cause chronic adverse health effects [23]. For all intents and purposes, the platform’s approach to content moderation appears to be working as promised in this setting.

Second, this exercise suggests that the use of the YouTube API’s *RelatedToVideoID* parameter and clean browsers are not valid replacements for real user experiences when studying the platform’s recommendation system. Real users searching for antivaccine content received less irrelevant content, and more provaccine content, than the first 2 automated approaches would suggest. Recommended videos suggested by YouTube’s API and through simulated browsers, as compared to users with real watch histories, are usually longer and more popular (as defined by higher view counts). Furthermore, the distribution of genres for recommended videos remained relatively constant as real users advanced through their search trajectories, whereas the YouTube API appears to have initially pushed more news and political content, and a clean browser encountered a larger share of educational content. These differences suggest that collecting data from real users of the platform may be well worth the additional effort for researchers seeking to understand the recommendations made by the algorithm in practice.

### Limitations

This study is not without limitations. First, while we aimed to design a simple and consistent approach to data collection, it is challenging to accurately characterize the YouTube recommender system using trajectories starting from a single seed video. This study focuses on testing a breadth of auditing mechanisms instead of a breadth of trajectory seeds. Future studies could examine other seed videos, for example, by starting trajectories with an antivaccine video.

Second, it is possible that we do not accurately classify whether content is pro- or antivaccine. Our algorithm may make mistakes in categorizing individual videos, and this problem may be exacerbated by antivaccine communities’ use of various strategies to evade content moderation and reach a larger audience. For example, there are work-arounds to avoid being flagged as “harmful content” on YouTube, such as using lexical variations (“that thing arrived in the United States”) or emphasizing concepts like “informed consent” and “health freedom.” Rather than standard prerecorded video content, they may also use live streams and antivaccine advertisements to convey their message [45]. Therefore, this study may underestimate the occurrence of antivaccine sentiment on YouTube.

Third, our 4 approaches to data collection are not entirely comparable. While real users (WHO infodemic managers, UN users, and MTurk workers) were given the task of actively looking for antivaccine content, trajectories collected with the YouTube API or through a clean browser proceeded by simply following the first recommendation at each step. Furthermore, the *RelatedToVideoID* parameter collected from the YouTube API is not explicitly a recommendation and is generated using a different algorithm altogether. Although each approach involves a slightly different data collection strategy, we attempted to select approaches that researchers might actually use in order to provide practical guidance on the differences between them.

The “targeted search” with which we tasked real users was designed to approximate real-world viewing behavior, in which people do not select videos at random but rather look for content that engages them. Nevertheless, it is interesting to note that real users were actually not very successful at this targeted search; a relatively low fraction of their trajectories ended in antivaccine content. This may reflect the lack of such content among platform recommendations, or the challenges faced by users when discriminating between pro- and antivaccine content.

Finally, we note that while YouTube appears to be effective at moderating antivaccine content in the United States, we cannot draw broader conclusions about other types of content and contexts. A previous study [46] revealed that COVID-19–themed videos in hard-hit countries used different messaging approaches and gained attention at different times. As the popularity of web content is usually driven by language similarity and geographic proximity [47,48], we cannot draw conclusions about the spread of content in other languages and countries. It is possible that vaccine-related misinformation is moderated more effectively in English than in other languages.

Encouragingly, regardless of which data collection strategy we use, we do not find evidence that YouTube’s recommendation algorithm promotes antivaccine content; if anything, the algorithm’s recommendations appear to favor other popular, health-related videos. In other words, in this context, the algorithm’s behavior is more consistent with a blockbuster strategy than a “rabbit hole” approach. However, we note that our understanding of the algorithm’s behavior varies significantly according to whether we study data from real users or attempt to collect synthetic data using simulated browsers or the YouTube API, suggesting that researchers interested in examining the platform’s recommendations should increase their efforts to collect data from actual users.

### Conclusions

This research was motivated by an interest in better understanding the actions of YouTube’s recommendation algorithm in the context of COVID-19 vaccine hesitancy so that the platform can be held accountable for its influence over viewing behavior. While YouTube appears to be successfully moderating antivaccine content, we also find that it promotes a large volume of irrelevant content. Importantly, we find that YouTube’s API and a simulated browser do not proxy actual viewing experiences, suggesting that data from real users are needed to better understand the workings of the platform.

## Abbreviations

API: application programming interface
CNN: convolutional neural network
GloVe: global vectors for word representation
IDF: inverse document frequency
LSTM: Long Short-Term Memory
Mturk: Amazon Mechanical Turk
SMOTE: synthetic minority oversampling technique
SVM: support vector machines
TF: term frequency
TF-IDF: term frequency-inverse document frequency
UN: United Nations
WHO: World Health Organization

## Data Availability

The data sets generated during or analyzed during this study are not publicly available as they contain user video browsing information and therefore might have privacy implications. However, aggregated data can be obtained from the corresponding author on reasonable request.
